# A Novel Co-Culture Model Reveals Enhanced CFTR Rescue in Primary Cystic Fibrosis Airway Epithelial Cultures with Persistent *Pseudomonas aeruginosa* Infection

**DOI:** 10.3390/cells12222618

**Published:** 2023-11-13

**Authors:** Deborah M. Cholon, Matthew A. Greenwald, Matthew G. Higgs, Nancy L. Quinney, Susan E. Boyles, Suzanne L. Meinig, John T. Minges, Ashlesha Chaubal, Robert Tarran, Carla M. P. Ribeiro, Matthew C. Wolfgang, Martina Gentzsch

**Affiliations:** 1 Marsico Lung Institute and Cystic Fibrosis Research Center, School of Medicine, University of North Carolina, Chapel Hill, NC 27599, USA; deborah_cholon@med.unc.edu (D.M.C.); matthew_greenwald@med.unc.edu (M.A.G.);; 2 Department of Microbiology and Immunology, University of North Carolina, Chapel Hill, NC 27599, USA; 3 Pharmaceutical Product Development (PPD), Thermo Fisher Scientific, Morrisville, NC 27560, USA; 4 Division of Genetic, Department of Internal Medicine, Environmental and Inhalational Disease, University of Kansas Medical Center, Kansas City, KS 66160, USA; 5 Division of Pulmonary Diseases, Department of Medicine, School of Medicine, University of North Carolina, Chapel Hill, NC 27599, USA; 6 Department of Cell Biology and Physiology, School of Medicine, University of North Carolina, Chapel Hill, NC 27599, USA; 7 Division of Pediatric Pulmonology, Department of Pediatrics, School of Medicine, University of North Carolina, Chapel Hill, NC 27599, USA

**Keywords:** CFTR, cystic fibrosis, airway epithelia, *Pseudomonas aeruginosa*, cytokines, CFTR modulators

## Abstract

People with cystic fibrosis (pwCF) suffer from chronic and recurring bacterial lung infections that begin very early in life and contribute to progressive lung failure. CF is caused by mutations in the CF transmembrane conductance regulator (*CFTR*) gene, which encodes an ion channel important for maintaining the proper hydration of pulmonary surfaces. When CFTR function is ablated or impaired, airways develop thickened, adherent mucus that contributes to a vicious cycle of infection and inflammation. Therapeutics for pwCF, called CFTR modulators, target the CFTR defect directly, restoring airway surface hydration and mucociliary clearance. However, even with CFTR modulator therapy, bacterial infections persist. To develop a relevant model of diseased airway epithelium, we established a primary human airway epithelium culture system with persistent *Pseudomonas aeruginosa* infection. We used this model to examine the effects of CFTR modulators on CFTR maturation, CFTR function, and bacterial persistence. We found that the presence of *P. aeruginosa* increased CFTR mRNA, protein, and function. We also found that CFTR modulators caused a decrease in *P. aeruginosa* burden. These results demonstrate the importance of including live bacteria to accurately model the CF lung, and that understanding the effects of infection on CFTR rescue by CFTR modulators is critical to evaluating and optimizing drug therapies for all pwCF.

## 1. Introduction

Cystic fibrosis transmembrane conductance regulator (CFTR) is an ion channel that mediates chloride and bicarbonate transport across epithelial cells, to control mucosal surface hydration and airway surface liquid (ASL) volume. In people with CF (pwCF), mucosal surfaces become dehydrated, and mucus viscosity is increased, which leads to airway plugging and decreased mucociliary clearance (MCC), resulting in increased inflammation and susceptibility to both bacterial and viral pathogens [1,2,3]. In CF, mucus accumulation in the lungs precedes bacterial infection [4]. *Staphylococcus aureus* infection is common in younger pwCF followed by the increasing prevalence of *P. aeruginosa*, which becomes the dominant pathogen in late-stage disease [5,6]. The establishment of chronic bacterial infection is associated with worse clinical outcomes and reduced antibiotic efficacy [7,8].

Therapies to enhance airway hydration have been developed and approved by regulatory agencies in the US and EU, i.e., small-molecule CFTR modulators that act directly on the mutant CFTR protein to restore its activity. These CFTR modulators are categorized as correctors, which promote the transfer of mutant CFTR to the apical membrane, and potentiators, which increase CFTR channel activity at the cell surface. These CFTR modulators can be used alone or in combination for pwCF, depending on the class of CFTR mutations. The potentiator ivacaftor(I)/VX-770 is the active component of the drug Kalydeco, which is useful for pwCF with CFTR gating mutations, such as G551D [9,10]. VX-770 combined with corrector lumacaftor/VX-809 or with corrector tezacaftor(T)/VX-661 are the active components of Orkambi or Symdeko/Symkevi, respectively. These combination therapies are beneficial for pwCF that are homozygous for the most common CFTR mutation, F508del, which is a folding/processing mutation [11,12,13]. Orkambi and Symdeko/Symkevi are not as beneficial for the F508del mutation as Kalydeco is for gating mutations due to the degradation of F508del CFTR by VX-770 [14,15]. Therefore, the next-generation correctors were developed: bamocaftor/VX-659 and elexacaftor(E)/VX-445. The combination of VX-445(E)/VX-661(T)/VX-770(I) represents the active components of triple therapy, ETI, or Trikafta/Kaftrio, which is highly beneficial for pwCF with F508del and other CFTR mutations, which accounts for ~90% of pwCF [16,17,18]. As Kalydeco and Trikafta/Kaftrio are more efficacious for pwCF, they are now known as highly effective modulator therapies (HEMTs) [19].

PwCF treated with HEMTs experience marked clinical improvements in lung function (FEV_1_), reduced inflammatory markers, decline in pulmonary exacerbations, and improved quality of life. VX-770 enhances CFTR activity by approximately 50% in vitro in electrophysiology experiments using primary human bronchial epithelial (HBE) cultures expressing the CFTR gating mutation, G551D [20,21]. In vivo, pwCF with G551D CFTR taking VX-770 showed a 10% improvement in their FEV_1_, a 55% reduction in pulmonary exacerbations, increased weight gain, decreased respiratory symptoms, and substantially reduced sweat chloride [19,22]. These in vitro and in vivo results have become the benchmark for HEMT for rescuing CFTR [19]. VX-770 also resolves mucus plugging and MCC [23,24,25], reduces bronchiectasis and sinus disease severity [26,27,28,29,30,31,32], and may also improve mucosal function in the gut [25,33] and insulin secretion in CF-related diabetes [34,35,36]. Based on in vitro data in HBE cultures, F508del CFTR protein maturation and chloride transport significantly improved upon treatment with ETI [37]. In pwCF with one copy of F508del CFTR, ETI improved FEV_1_ by 14.3%, reduced the pulmonary exacerbation rate by 63%, and improved respiratory symptoms. In pwCF homozygous for F508del, ETI increased FEV_1_ by 10% above VX-661/VX-770 and improved respiratory symptoms [16,17]. Current in vitro and in vivo data indicate that ETI exceeds the HEMT benchmark set by the VX-770 treatment of pwCF with G551D and other gating mutations [19].

Despite the clear benefits of CFTR modulators in pwCF, the impact on pre-existing bacterial infection appears limited and transitory. PwCF with G551D CFTR mutations showed a decrease in *P. aeruginosa* in sputum samples during the first year of taking VX-770/Kalydeco; however, infection is not eradicated and pathogen burden subsequently rebounded at 6 and 12 months after an initial decline [38]. Several smaller studies evaluating the microbiological outcomes in individuals taking Kalydeco showed a reduction in *P. aeruginosa*-positive cultures, but no significant decrease in *S. aureus* [39]. Even in pwCF taking the ETI triple therapy, bacterial infections persisted after an initial improvement [40]. A longitudinal study of pwCF taking ETI also demonstrated that bacterial infections endure and need to be controlled by additional therapies [41]. Thus, CFTR modulators are limited in reducing chronic bacterial lung infection, which is the main cause of the pulmonary tissue damage and progressive respiratory insufficiency that lead to arduous daily therapies, limited quality of life, and premature death in pwCF [38,40]. More studies are needed to elucidate how pre-existing bacterial infection and inflammation affect the efficacy of CFTR-targeted therapies [42].

Prior studies found that *P. aeruginosa* reduced CFTR chloride secretion in airway epithelial cells [43,44,45]. However, these studies were conducted with a relatively short infection time. A small number of reports have utilized long-term co-cultures of airway epithelial cells with bacteria, which were conducted in cell lines [46,47]. Here, we examine the efficacy of CFTR modulator therapies in primary CF airway epithelial cultures with prolonged luminal infection. The results obtained from this study demonstrate the importance of infection and inflammation in drug pharmacology and efficacy when optimizing therapeutics for pwCF.

## 2. Materials and Methods

### 2.1. Epithelial and Bacterial Co-Culture

HBE cells containing primarily large airway cells from proximal sites from adult male and female donors were seeded on 12 mm Millicell Cell Culture Inserts (Millipore, Burlington, MA, USA) and maintained at the air–liquid interface (ALI) using a modified Lonza differentiation medium that includes a 1:1 ratio of BEBM (Lonza, Walkersville, MD, USA) and DMEM (Thermo Fisher Scientific, Waltham, MA, USA) plus BEGM SingleQuots (Lonza) as described elsewhere [48] for 3–4 weeks to allow cells to become fully differentiated as previously described [49,50]. All CF donors were homozygous for F508del CFTR. At least one week before the addition of bacteria, HBE cultures were switched to antibiotic-free medium and apical secretions were allowed to accumulate and form a mucus layer. Wild-type *P. aeruginosa* laboratory strain PAO1 was used for all experiments unless otherwise noted. *P. aeruginosa* was grown overnight at 37 °C in LB medium (Thermo Fisher) and then sub-cultured and grown until mid-exponential growth (assessed by optical density at 600 nm) was achieved. Bacteria were then washed and resuspended in HBE culture medium without antibiotics and added apically to HBE cultures (1 × 10^6^ bacteria in 5 µL). Except where otherwise noted, tobramycin (58 µg/mL; serum Cmax) was added to the basolateral media 3 h after the addition of bacteria. After 24 h of infection, the apical HBE surface was gently washed with 200 µL of culture medium to collect bacteria. Bacteria were serially diluted and plated on LB agar to enumerate bacterial burden. The integrity of HBE cultures was determined by measuring transepithelial resistance by EVOM2 or Ussing chambers. Epithelial cultures were further processed, as described below.

### 2.2. Drug Treatments

A CFTR corrector cocktail containing 2 correctors, either VX-659 (1 µM) or VX-445 (3 µM) plus VX-661 (10 µM) (Selleck Chemicals LLC, Houston, TX, USA), was applied to HBE cultures on the basolateral side chronically for 24 h. Potentiator VX-770 (1 µM) was added to the apical side of cultures for 30 min during Ussing chamber measurement or 30 min prior to lysing for Western blot or mRNA analysis, to mimic acute VX-770 treatment.

### 2.3. Antimicrobial Activity Assays

The direct antimicrobial activity of CFTR correctors was assessed by *P. aeruginosa* growth in HBE culture media. Here, 1 × 10^6^ CFU/mL of mid-exponential phase *P. aeruginosa* was inoculated into HBE cell culture medium in the absence or presence of CFTR modulators at the concentrations described above or vehicle (DMSO) control and cultured for 24 h at 37 °C. CFTR modulator synergy with tobramycin was assessed by broth microdilution according to Clinical Laboratory Standards Institute (CLSI) guidelines [51] in cation-adjusted Mueller–Hinton broth (MHB).

### 2.4. Western Blot Analysis

HBE in the presence and absence of bacteria were lysed after bacteria were washed off and CFTR proteins were immunoprecipitated and subjected to Western blot analysis using primary monoclonal antibodies specific for CFTR protein as previously described [14]. Briefly, whole-cell lysates of fully differentiated HBE cultures were prepared, and then CFTR was immunoprecipitated using rabbit anti-CFTR polyclonal antibody 155 (1:200; generously provided by Dr. John R. Riordan)). Samples were separated on 4 to 20% gradient SDS–polyacrylamide gel electrophoresis gels (Bio-Rad, Hercules, CA, USA) and then transferred to nitrocellulose. Blots were probed with mouse monoclonal anti-CFTR antibodies 596 and 217 (1:2000 each; CFTR Antibody Distribution Program) and then with Alexa Fluor 680-conjugated goat anti-mouse secondary antibody (1:10,000; Thermo Fisher). Rabbit anti-actin antibodies (1:4000; Cell Signaling, Beverly, MA, USA) were used as a loading control and detected using Alexa Fluor 790-conjugated goat anti-rabbit secondary antibody (1:20,000; Thermo Fisher). Protein bands were visualized using a Sapphire Biomolecular Imager (Azure Biosystems, Dublin, CA, USA) and bands were quantitated using AzureSpot imaging software (version 2.2.170, Azure Biosystems).

### 2.5. Ussing Chamber Measurements

HBE cultures were mounted in Ussing chambers to measure I_SC_ as previously described [14]. Prior to CFTR activation, the epithelial sodium channel (ENaC) activity was assessed by addition of amiloride (Amil). CFTR function was measured by acute addition of forskolin (Fsk) and VX-770 to activate CFTR, and CFTR inhibitor (CFTRinh-172), to demonstrate the specificity of the CFTR channel activity. UTP was added to activate the calcium-activated chloride channel (CaCC, also termed TMEM16A).

### 2.6. Microscopy

HBE cultures infected with PAO1 expressing GFP were mounted in an Attofluor chamber (Thermo Fisher) and imaged by confocal microscopy using a Leica SP8 confocal microscope equipped with a 63 × 1.2 NA glycerol objective lens. Then, 10 kDa dextran conjugated to Alexa 647 dye (Thermo Fisher) was used to visualize the apical airway surface liquid (ASL). Alexa 647 and GFP-*P. aeruginosa* were excited using 633 nm and 488 nm lasers, respectively. Images were captured along the surface of the culture (x-y axis) and then rendered to create an orthogonal view.

### 2.7. mRNA Analysis

Epithelial cultures with and without bacterial infection were frozen at −20 °C in QIAzol lysis reagent (Qiagen Sciences, Germantown, MD, USA). The cultures were subsequently treated with chloroform and total RNA was extracted using the RNeasy mini kit (Qiagen), as per the manufacturer’s protocol. RNA was eluted in Ambion^®^ DEPC-treated nuclease-free water. cDNA was synthesized using Bio-Rad iScript reverse transcriptase. A 1:20 dilution of cDNA was mixed with Bio-Rad SsoAdvanced supermix and Taqman probe sets for CFTR (Thermo Fisher) and the housekeeping gene TBP (Thermo Fisher). qPCR was run on a QuantStudio 6 (Thermo Fisher). CFTR gene expression data were expressed as the fold change of experimental samples over control samples (3 lung donors; *n* = 4 measurements per treatment), as reported [52].

### 2.8. Cytokine Analysis

The basolateral media from bacteria-infected and uninfected cultures were diluted and subjected to ELISA to determine the concentration of IL-6 and IL-8 in each sample. The basolateral medium was sampled rather than the apical fluid, as differences in apical ASL volume between the different treatment groups would introduce bias in the cytokine measurement from the apical ASL. The samples were evaluated with the following ELISA kits (R&D Systems, Minneapolis, MN, USA): Ancillary kit (DY008), using a 1:10 dilution of media with the IL-6 kit (DY206), and a 1:50 dilution of media with the IL-8 kit (DY208). ELISA data were captured using a CLARIOstar plate reader (BMG Labtech, Cary, NC, USA). Values were expressed as cytokine concentrations (pg/mL). Each condition was measured in biological triplicate and samples for ELISA were evaluated in technical duplicate, as previously described [52].

### 2.9. Statistics

Results are presented as means of average responses per primary HBE cell donor, and error bars show SEM. Statistical analyses were performed by one-way analysis of variance (ANOVA) or two-tailed Student’s *t*-test in GraphPad Prism (version 9.5.0, GraphPad Software, Boston, MA, USA). *p* values of <0.05 indicate statistical significance.

## 3. Results

### 3.1. Model of Persistent P. aeruginosa Airway Infection Using Well-Differentiated Primary HBE Cultures

**Figure 1:**
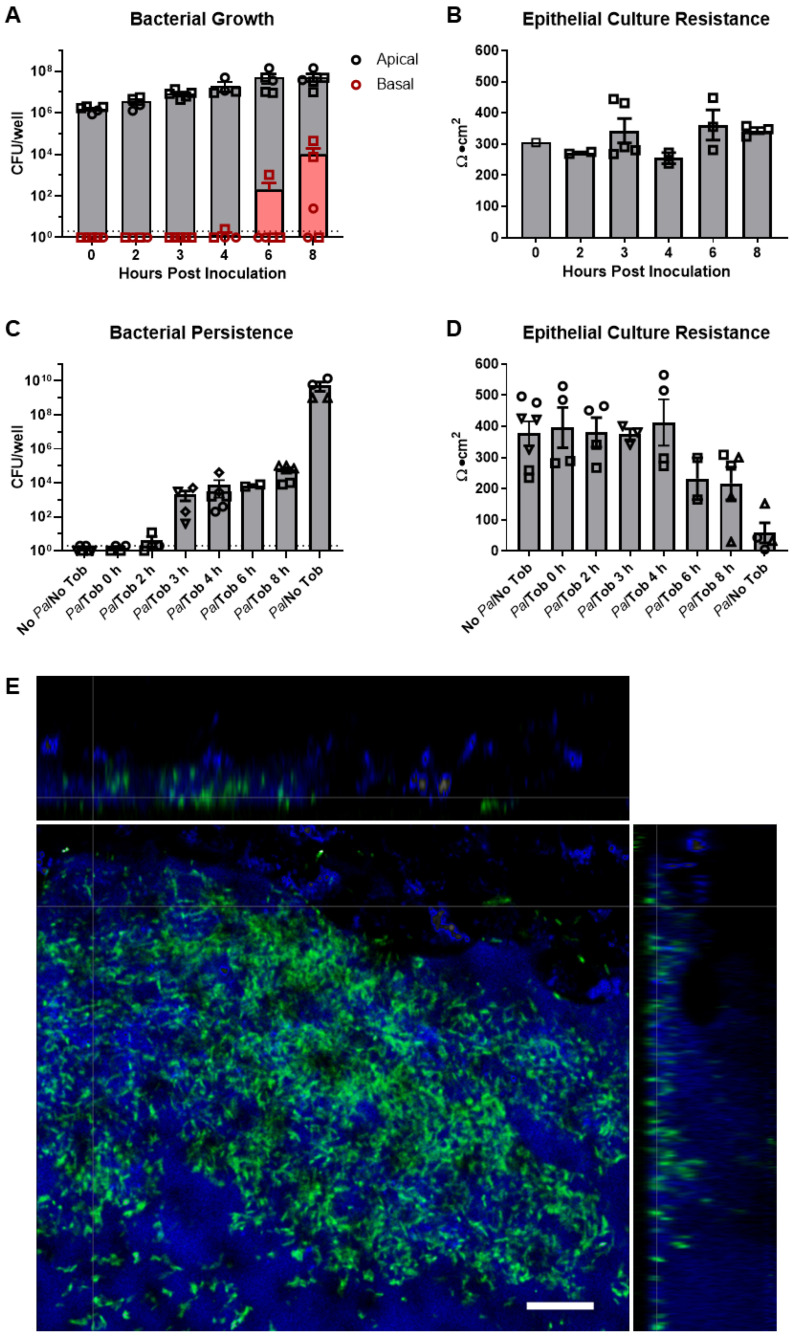
Novel method for co-culturing *P. aeruginosa* with primary HBE cells. Primary HBE cultures from multiple donors were grown at ALI and infected apically with *P. aeruginosa* (*Pa*) strain, PAO1 (1 × 10^6^ CFU), and allowed to grow with or without the presence of basolateral tobramycin (Tob; 58 µg/mL) added at different time points relative to the addition of *Pa*. (**A**) Growth kinetics of *Pa* from the apical mucus washings and infiltration into the basolateral culture media (2 donors, *n* = 2–5 per donor; limit of detection = 2 CFU/well). (**B**) The integrity of HBE cultures was determined by measuring transepithelial resistance by EVOM2 (*n* = 2–5 per donor). (**C**) Twenty-four hours after infection, *Pa* was collected from apical washes and colony counts were obtained (5 donors, *n* = 2–3 per donor; limit of detection = 2 CFU/well). (**D**) The integrity of HBE cultures was determined by measuring resistance in Ussing chambers (*n* = 2–3 per donor). (**E**) *P. aeruginosa* detected in epithelial ASL. Green = *P. aeruginosa* (GFP), blue = ASL (Dextran, Alexa Fluor™ 647; Thermo Fisher). Images were captured along the surface of the HBE culture (x-y axis), and then rendered to create the x–z orthogonal views (top and right). Scale bar = 50 µm.

The in vitro co-culturing of *P. aeruginosa* on HBE cells has proven challenging due to unmitigated bacterial growth resulting in rapid host tissue destruction, an artifact of a closed system that is not observed in the in vivo environment. Thus, current co-culture models have been limited to short infection periods, typically ≤6 h, and primarily carried out in immortalized cell lines [43,53,54,55]. We previously found that *P. aeruginosa* could invade the epithelial layer of well-differentiated primary HBE cultures grown at the air–liquid interface without a mucus layer in as little as 3 h [56]. However, lung infection in pwCF is largely limited to luminal mucus, and bacteria are rarely observed to physically interact with the epithelial surface [57,58]. To better replicate human infection, we allowed fully differentiated HBE cultures to accumulate a mucus layer for a minimum of 7 days prior to infection. We found that in primary well-differentiated non-CF HBE cultures, the mucus layer reduced tissue penetration and damage; however, basolateral dissemination could be detected after 4 h (Figure 1A, red symbols), despite the maintenance of transepithelial resistance for at least 8 h (Figure 1B). The timing of the basolateral dissemination is similar to what was previously observed in cell lines in which transepithelial resistance decreased after 5 h of bacterial infection [59]. While the mucus layer appeared to delay tissue damage and more accurately reflects the site of native infection, bacterial growth was largely uncontrolled with apical *P. aeruginosa* burden increasing >1.5-logs in 8 h (Figure 1A, black symbols), indicative of an acute infection.

In order to create a more persistent infection model, apically infected HBE cultures were treated basally with tobramycin at the serum Cmax, 58 μg/mL [60], at different time points post infection. We then determined whether antibiotic exposure could control bacterial growth and prevent epithelial culture damage, thereby extending the infection window. Tobramycin is the most widely used antipseudomonal antibiotic by pwCF, and a relevant feature of the chronic infection environment [61]. After 24 h, *P. aeruginosa* was collected from the apical surface, it was washed to remove residual antibiotics, and the bacteria were enumerated (Figure 1C). The addition of tobramycin to the basal media immediately after apical infection (*Pa*/Tob 0 h) or 2 h post infection (*Pa*/Tob 2 h) generally eradicated the infection. When tobramycin was added basally 3–8 h after infection, viable bacteria could be recovered in apical washes 24 h later. Due to the high concentration of tobramycin in the basal compartment, basolateral dissemination could not be directly measured. However, the integrity of HBE cultures was determined by measuring transepithelial resistance in Ussing chambers 24 h after infection (Figure 1D). Transepithelial resistance measurements indicated that the integrity of the infected HBE cultures remained intact during infection, similar to uninfected cultures (No *Pa*/No Tob), when the antibiotic was added at or before 4 h post infection. When the antibiotic was added 6 or 8 h post-infection, a decrease in resistance was detected after 24 h, although some level of culture integrity was maintained compared to control cultures in which antibiotics were not added post-infection (*Pa*/No Tob). These data demonstrate the feasibility of maintaining primary HBE and bacterial co-cultures for longer time periods than previously achieved. Based on variability in the invasion of *P. aeruginosa* into the basolateral media beginning as early as 4 h (Figure 1A), the addition of tobramycin 3 h post-infection was selected as a conservative but reproducible method to generate prolonged infection for 24 h. Using these conditions, we visualized HBE cultures infected with a PAO1 strain expressing GFP for 24 h by high-resolution confocal microscopy (Figure 1E). *P. aeruginosa* appeared to reside within the mucus-laden ASL and form aggregate or biofilm-like community structures consistent with those observed in CF sputum and airway sections [62,63].

### 3.2. CFTR Correctors Caused a Decrease in P. aeruginosa Burden When Co-Cultured with HBE Cells

**Figure 2:**
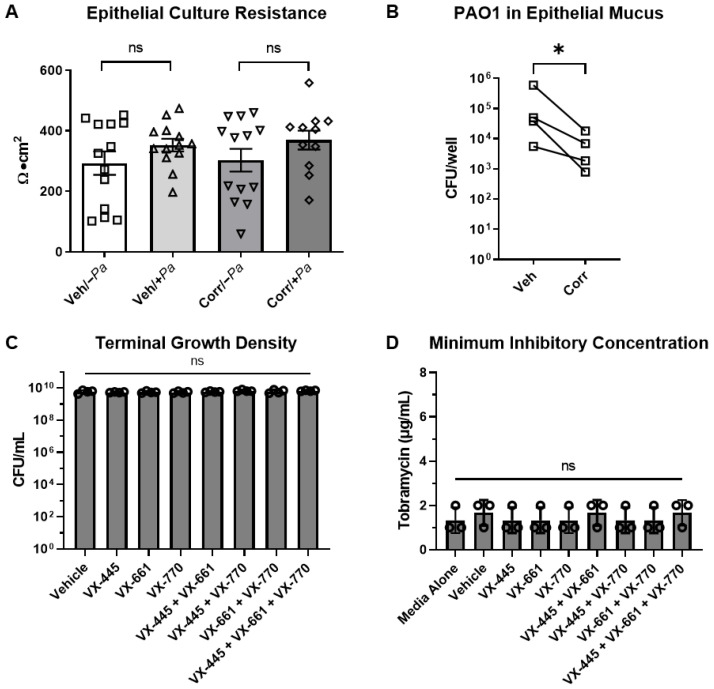
CFTR correctors caused a decrease in *P. aeruginosa* burden when co-cultured with HBE. (**A**,**B**) Primary CF HBE cultures (F508del/F508del) were treated with 58 µg/mL tobramycin 3 h after *P. aeruginosa* PAO1 infection (+*Pa*) or mock infection with media alone (−*Pa*). Infection/mock infection was for 24 h. Correctors (Corr) VX-661 (10 µM) and VX-659 (1 µM) or VX-445 (3 µM) were added basolaterally at the time of infection. (**A**) Epithelial cultures remained intact 24 h after *P. aeruginosa* infection, as measured by resistance in Ussing chambers (4 donors, *n* = 2–4 per donor). (**B**) *P. aeruginosa* burden from apical washings from vehicle or corrector-rescued CF HBE cultures. *P. aeruginosa* burden was decreased in the presence of CFTR correctors. Data points represent the average for each HBE culture donor (4 donors, *n* = 5–6 per donor). (**C**,**D**) Direct treatment (no HBE) of *P. aeruginosa* with CFTR modulators and tobramycin. (**C**) *P. aeruginosa* growth in cell culture medium in the presence of vehicle (DMSO) or CFTR modulators individually and in combination. (**D**) Minimum inhibitory concentration (MIC) of *P. aeruginosa* to tobramycin in the absence or presence of CFTR modulators or vehicle control (DMSO). Statistical significance was determined by one-way ANOVA with Dunnet’s multiple comparison, * *p* < 0.05, ns = not significant.

Using our co-culture method, we next evaluated the impact of CFTR correctors on bacterial growth. To achieve this, we infected primary HBE cultures derived from CF donors (F508del/F508del) with *P. aeruginosa*, as described above. Infected cultures were treated with vehicle or CFTR correctors, VX-661 and VX-659 or VX-445 (Selleck Chemicalsfor 24 h (Figure 2). The presence of correctors and bacteria did not affect epithelial culture resistance (Figure 2A); however, the presence of correctors led to a decrease in bacterial burden compared to untreated cultures (Figure 2B). This suggests that CFTR correctors may inhibit *P. aeruginosa* growth directly or indirectly through the restoration of CFTR function.

To evaluate the direct antimicrobial activity of the CFTR modulators or possible synergy with tobramycin in the absence of HBE, we performed terminal growth assays and tobramycin minimum inhibitory concentration (MIC) assays with *P. aeruginosa* in the presence or absence of each CFTR modulator compound individually and in combination (Figure 2C,D). As previously described, VX-770 did not exhibit antimicrobial activity against *P. aeruginosa* or synergy with tobramycin, as evidenced by the lack of a shift in the MIC [64]. Furthermore, VX-445 and VX-661, alone or in combination, did not show any indication of antimicrobial activity or synergy with tobramycin (Figure 2C,D).

### 3.3. The Presence of P. aeruginosa Enhanced CFTR Protein Maturation, mRNA, and Function

**Figure 3:**
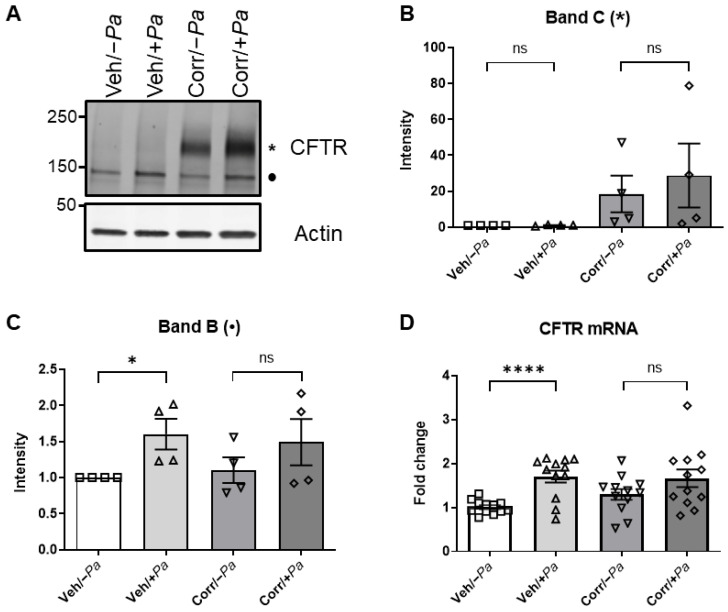
*P. aeruginosa* caused an increase in F508del CFTR protein maturation and mRNA in CF HBE cultures. After *P. aeruginosa* (or mock infection) was washed from the apical side, HBE cells were processed to analyze CFTR protein levels or mRNA. (**A**) CFTR was immunoprecipitated and then subjected to Western blot analysis using antibodies specific for CFTR. Actin was used as a loading control. Representative Western blot showing substantial increase in CFTR levels in corrector-rescued F508del CFTR when infected with *P. aeruginosa*. Quantitations of F508del CFTR mature band C (*) (**B**) and immature band B (•) (**C**) are shown. Values were normalized to Veh/−*Pa* (*n* = 4 donors, * *p* < 0.05). (**D**) mRNA was extracted from treated and untreated HBE cultures and then quantitated by qRT-PCR (3 donors, *n* = 4 per donor, **** *p* < 0.0001; ns = not significant).

We next examined how *P. aeruginosa* infection affected the function and efficacy of the CFTR correctors. Specifically, we measured the levels of mature and immature CFTR protein following the treatment of HBE cultures (F508del/F508del) with correctors in the presence or absence of *P. aeruginosa* infection. After 24 h, HBE cultures were lysed, the CFTR protein was immunoprecipitated, and samples were subjected to Western blot analysis to detect and quantify the amounts of mature and immature F508del CFTR (Figure 3A–C). The mature, complex-glycosylated, higher-molecular-weight band of CFTR (band C, *; Figure 3A) was quantitated (Figure 3B). As expected in the presence of correctors, the mature F508del CFTR band C, * was detectable (Figure 3A,B; Corr/−*Pa*). Although it was not statistically significant, in the presence of *P. aeruginosa* there was an increase in signal intensity of corrector-rescued CFTR band C (Figure 3A,B; Corr/+*Pa*). The immature, lower-molecular-weight band of CFTR (band B, •; Figure 3A) was also quantitated (Figure 3C) and shows that *P. aeruginosa* infection caused an increase in immature F508del CFTR levels that are significantly different in the absence of correctors. After the bacterial infection of HBE, epithelial cultures were processed for CFTR mRNA quantitation by qRT-PCR (Figure 3D). In the absence of correctors, the presence of *P. aeruginosa* (Veh/+*Pa*) caused a significant increase in CFTR mRNA compared to mock infected (Veh/−*Pa*). This result parallels the amount of immature CFTR band B that is formed (Figure 3C). These results suggest that despite an increase in total CFTR protein, the presence of *P. aeruginosa* enhances mature CFTR in the presence of correctors.

**Figure 4:**
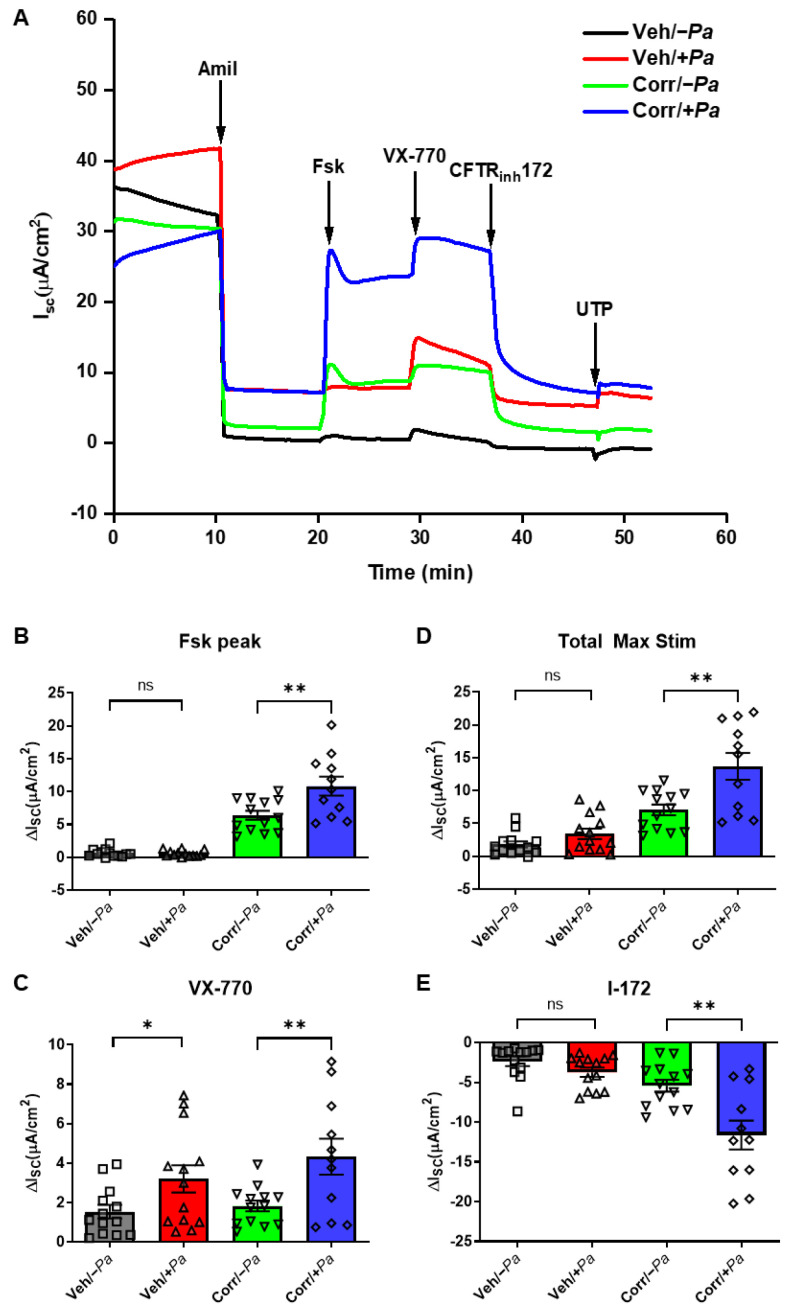
*P. aeruginosa* led to an increase in F508del CFTR function in CF HBE cultures. Ussing chamber analyses to determine the effects of *P. aeruginosa* on F508del CFTR rescue. (**A**) Representative traces in HBE from a CF (F508del/F508del) donor. Quantitation of short-circuit current (I_sc_) responses to forskolin (Fsk; (**B**)) and VX-770 (VX-770; (**C**)); the total maximum stimulation from both forskolin and VX-770 (Total Max Stim) (**D**) and CFTR inhibitor-172 (I-172; (**E**)) are shown (4 donors, *n* = 2–4 per donor, * *p* < 0.05, ** *p* < 0.01, ns = not significant). *P. aeruginosa* caused a significant increase in I_sc_ in all acute Ussing chamber treatments shown (**B**–**E**) with corrector-treated HBE. In the absence of correctors, only acute VX-770 caused a significant increase in I_sc_ (**C**).

Bacterial–epithelial co-cultures were also used to determine the CFTR function in Ussing chambers that measure transepithelial ion transport (Figure 4). Representative traces are shown in Figure 4A. Short-circuit currents (I_SC_) of corrector-rescued F508del CFTR in response to forskolin increased significantly when *P. aeruginosa* was present (Figure 4B), indicating an increase in CFTR activation with correctors plus *P. aeruginosa*. Acute treatment with VX-770 led to a significant increase in I_SC_ when *P. aeruginosa* was present with and without correctors (Figure 4C), demonstrating enhanced CFTR potentiation in the presence of *P. aeruginosa* that is independent of corrector treatment. The total maximum stimulation with both forskolin and VX-770 (Total Max Stim) showed a slight but non-significant increase in I_SC_ in cultures without correctors when *P. aeruginosa* was present and a robust and significant increase in corrector-treated samples when *P. aeruginosa* was present (Figure 4D). Treatment with CFTR inhibitor-172 (I-172) resulted in decreases in I_SC_ in a similar pattern to Figure 4D (Figure 4E), indicating that the I_SC_ changes are due to enhanced CFTR activity, not other ion channels. Most importantly, there was a significant, approximately 2-fold increase in the amount of rescued F508del CFTR function when *P. aeruginosa* was present. Thus, 24 h of infection with *P. aeruginosa* can enhance F508del CFTR mRNA levels, protein maturation, and function in CF airway epithelial cultures.

### 3.4. P. aeruginosa Infection Increased Activity of CaCC in HBE Cultures

**Figure 5:**
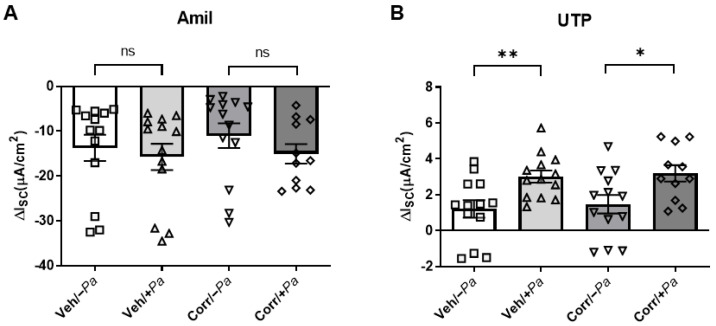
*P. aeruginosa* infection significantly increases UTP responses in HBE cultures. Ussing chamber analyses to determine the effects of *P. aeruginosa* infection on other ion channels. (**A**) I_sc_ responses to amiloride (Amil) showed a nonsignificant increase in the inhibition of ENaC in the presence of *P. aeruginosa*. (**B**) I_sc_ responses to UTP demonstrate a significant increase in CaCC activity (4 donors, *n* = 2–4 per donor, * *p* < 0.05, ** *p* < 0.01, ns = not significant).

To understand the effects of bacterial infection on other ion channels, we measured the activity of ENaC and CaCC in HBE cultures in the presence or absence of *P. aeruginosa* infection in Ussing chambers (Figure 5). Amiloride was added to inhibit ENaC (Figure 5A), and UTP was added to activate CaCC (Figure 5B). While there was no significant change in ENaC function (I_SC_) in the presence of *P. aeruginosa,* there was a significant increase in CaCC activity. These data suggest that the effect of *P. aeruginosa* infection is selective in influencing ion channel activity for CFTR and CaCC, but not ENaC.

### 3.5. Cytokine Secretion Is Increased upon Bacterial Infection

**Figure 6:**
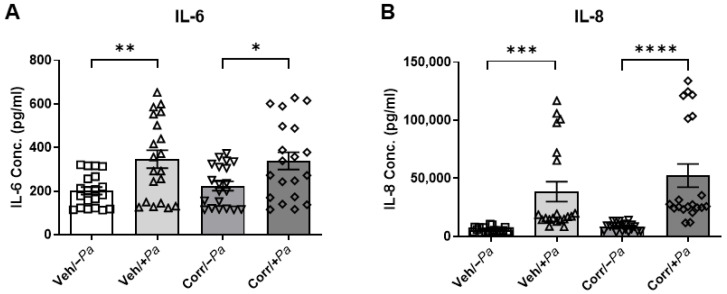
*P. aeruginosa* caused an increase in the secretion of cytokines IL-6 and IL-8. Basolateral media from treated and untreated HBE cultures were diluted and subjected to ELISA to determine the amounts of cytokines released (3 donors, *n* = 6–8 per donor, * *p* < 0.05, ** *p* < 0.01, *** *p* < 0.001, **** *p* < 0.0001). Infection with *P. aeruginosa* caused significant increases in IL-6 (**A**) and IL-8 (**B**) secretion, which was independent of correctors.

The effects of bacterial infection on cytokine secretion in this co-culture model were examined. Media from the basolateral side of HBE cultures, treated with or without correctors and infected with *P. aeruginosa* or mock infected, were subjected to ELISA to determine the release of cytokines IL-6 and IL-8. IL-6 and IL-8 represent inflammatory markers relevant to CF airways. There was a significant increase in IL-6 (Figure 6A) and IL-8 (Figure 6B), independent of corrector-rescue, in *P. aeruginosa*-infected HBE, indicating that *P. aeruginosa* induced inflammation in our co-culture model. Interestingly, IL-6 and IL-8 levels were similar in infected cultures regardless of CFTR corrector treatment, which was associated with a reduction in 24 h *P. aeruginosa* burdens (Figure 2B).

## 4. Discussion

Despite the availability of highly effective modulator therapy, *P. aeruginosa* remains a major cause of lung infections in pwCF [38,40]. The results of our study demonstrate the feasibility of co-culturing primary HBE with *P. aeruginosa* for extended time periods in order to examine the effects of persistent bacterial infection on airway epithelia. This is important for understanding the effects of chronic bacterial infection on host response, novel drug interactions, and specifically the ability of CFTR modulators to rescue mutant CFTR. It is also a useful model for elucidating the mechanism underlying the decrease in bacterial burden upon the rescue of CFTR in pwCF. This model could support studies to examine why bacteria persist and resurge in pwCF despite CFTR modulator therapy and improved clinical metrics. Furthermore, based on recent studies, this model can be used to deepen our understanding of how phenotypic and genotypic variations in *P. aeruginosa* clinical isolates can affect lung disease progression and severity [65,66].

While cell lines are a suitable tool for many CFTR studies, there are drastic differences in the responses of immortalized cell lines versus primary airway epithelial cells in response to bacterial exposure. We utilized primary HBE cultures infected with live, metabolically active *P. aeruginosa* to more accurately mimic the chronically infected airway epithelia in the lungs of pwCF. The co-culturing of *P. aeruginosa* on HBE cells has thus far been limited to short infection windows (typically < 8 h) due to the unrestricted growth of bacteria and damage to the epithelial layer in a closed system lacking innate immune defenses or other relevant treatments that limit bacterial growth. Such short infection periods leave little time to observe long-term host and bacterial adaptations, along with the remodeling that occurs in chronic infections. By restricting *P. aeruginosa* outgrowth with the addition of clinically relevant antibiotics in the basal media, we were able to achieve a prolonged infection for 24 h without the disruption of epithelial integrity or bacterial infiltration into the basolateral compartment. In pwCF, *P. aeruginosa* adapts to the mucus-dominated metabolic environment of the CF airways as the infection becomes persistent. Longer infection times likely allow *P. aeruginosa* more time to adapt to the host/mucus environment, and the remodeling of the host transcriptional responses. These temporal changes may begin to explain the discrepancy between experiments conducted by other groups with shorter, more acute infection times (4–6 h) [43,44,45,54,55,67], versus the current study with a more prolonged infection (24 h). This approach opens opportunities for future mechanistic and therapeutic studies in a relevant model system of persistent airway infection.

It is important to eradicate bacterial infections as early as possible, before chronic infection is established. PwCF typically have *S. aureus* infections earlier in life followed by subsequent *P. aeruginosa* infection; thus, experiments with HBE co-cultured with multiple species of bacteria should be conducted to determine the effects of HEMT and antibiotics on CFTR function and bacterial growth in the context of a polymicrobial infection. Additionally, ETI intervention earlier in life may be paramount in order to help prevent the onset of chronic bacterial infection.

In the current study, the presence of CFTR correctors decreased bacterial burden. This was not due to the direct antibiotic activity of the correctors or synergistic activity of correctors with tobramycin. However, an indirect effect of the correctors on bacterial survival through increased CFTR function, which will increase mucus hydration and pH, could create an environment that may be less conducive to bacterial persistence, perhaps by causing bacteria to be less tolerant to antibiotics [68], which remains a possible explanation. Additional studies are necessary to evaluate the relationship between CFTR correctors and bacterial persistence in epithelial cell cultures from pwCF. In addition, FDA safety information for ETI recommends that patients do not take ETI if they are taking certain antibiotics, e.g., rifampin. Rifampin has been shown to enhance the metabolic breakdown of VX-770, the CFTR potentiator component of ETI, thereby decreasing FEV_1_ in CF patients [69]. Because it is possible that other components of ETI can also be negatively impacted by antibiotics, future studies are needed to test the effects of antibiotics on corrector-dependent CFTR rescue.

The CFTR modulator VX-770 has structural similarity with quinolone antibiotics, and is thought to display some antimicrobial activity. To examine this, one study showed some antibiotic activity of VX-770 against *S. aureus*, but little efficacy against *P. aeruginosa* [64]. There was a synergistic killing of *P. aeruginosa* when VX-770 was used together with antibiotic polymyxin B [70] or ciprofloxacin [71], and of *S. aureus* and *Streptococcus* species when VX-770 was used together with tobramycin [72]. A recent study compared the ability of HEMTs VX-770 versus ETI to synergize with antibiotics, and found that ETI treatment did not enhance the activity of most antibiotics against *P. aeruginosa* clinical isolates, with the exception of polymyxin B. The additive effect of ETI with polymyxin B was also seen in bacterial isolates treated with VX-770 alone, indicating that the other correctors in ETI (VX-445 and VX-661) do not contribute to the antibiotic-enhancing activity of VX-770 [73].

The rescue of CFTR ion channel function by modulators may change the characteristics of CF mucus, which in turn may affect bacterial persistence. Mucus in healthy lungs is composed of approximately 98% water and 2% solids. In contrast, in CF lungs, the mucin is hyperconcentrated at >6% solids [74,75,76,77,78]. Indeed, CFTR modulator treatment in CF HBE cells decreased mucus concentration, relaxed mucus network ultrastructure, and improved mucus transport, thereby restoring normal mucus characteristics [77]. An advanced understanding of the mucus properties upon CFTR modulator treatment [77] will allow for the better characterization of the influence of CFTR function on bacterial survival.

Studies with immortalized airway cells have previously noted CFTR-dependent changes in transepithelial resistance [79,80]. However, consistent with previous studies of freshly isolated human airway epithelia, we did not detect major differences in transepithelial resistance between non-CF and CF HBE cultures, or between CF HBE cultures ± CFTR correction [81]. During the chronic infection of CF lungs with *P. aeruginosa*, the bacteria are found within the stagnant mucus plugs and plaques, rather than directly contacting the airway epithelia [57,58]. This observation was recapitulated in our system by our fluorescence microscopy data showing *P. aeruginosa* remaining in the ASL. As CFTR rescue increases the hydration of airway mucus, the ASL volume should increase, which can be measured by fluorescence microscopy intended to detect changes in ion and fluid transport [82,83]. This increase in mucus hydration may have a negative impact on bacterial survival. Utilizing our co-culture system, mechanistic studies into the effects of CFTR modulators on bacterial survival due to changes in mucus characteristics can be investigated, and will provide important considerations for optimizing therapies for pwCF and other lung diseases.

CF shares some pathophysiology with COPD, asthma, and non-CF bronchiectasis, such as mucus obstruction and airway infections. It is important to consider that CFTR modulators intended for pwCF could be repurposed for other muco-obstructive diseases [84,85], which currently lack effective therapies. The chronic presence of metabolically active bacteria existing in biofilms needs to be incorporated into preclinical models of diseased lungs for the more accurate testing of treatments that modulate ion transport in order to identify and optimize treatments. Differentiated HBE cells from COPD and asthma patients can also be used to examine the effects of bacterial infection and drug treatments for these diseases.

The increase in CFTR protein and function observed after 24 h of bacterial infection is consistent with our previous studies using supernatant of mucopurulent material (SMM) from the lungs of pwCF, which induced HBE inflammation [86]. We found that in HBE expressing F508del CFTR treated with CFTR correctors, exposure to SMM for 24 h enhanced CFTR maturation and function [87,88]. The hyperinflammation induced by SMM leads to increased protein synthesis and ER expansion [86], which may promote the proper folding of F508del CFTR [87,88]. Furthermore, the current study showed that the presence of *P. aeruginosa* led to an increase in F508del CFTR function upon acute treatment with VX-770, even without the addition of correctors, indicating that infection alone can enhance CFTR’s responses to potentiators. Infection and inflammation are tightly intertwined, such that infection triggers inflammation, and mucus accumulation and inflammation precede infection [4]. SMM is composed of both infectious byproducts and inflammatory factors; the contribution of infection versus inflammatory factors in enhancing CFTR function in HBE cells can be compared using a bacterial–epithelial co-culture model versus adding SMM to HBE cultures.

*P. aeruginosa* exacerbates inflammation in the CF airway. A study on cytokines in the airways of pwCF found that both IL-8 and IL-6 were significantly increased upon colonization with *P. aeruginosa* [89]. This is consistent with our data showing that *P. aeruginosa* caused an increase in IL-6 and IL-8 in HBE cultures. Another study showed that IL-6 was important for controlling *P. aeruginosa* early in infection [90]. As inflammatory cytokines (IL-1, IL-6, IL-8, TNF-α) are induced by the infection of CF lungs [56,91,92,93], additional studies, e.g., bulk RNA sequencing, may reveal new inflammatory pathways that are upregulated by *P. aeruginosa* during prolonged infection of HBE. Our findings that inflammation persisted despite a reduced bacterial burden in the presence of CFTR correctors indicate that the infection induces some lasting effect, perhaps lasting damage that is not related to epithelial culture resistance. Alternatively, inflammation could persist due to a lack of bacterial clearance.

In summary, we developed a bacteria–epithelia co-culture model useful for studying persistent bacterial infection and the effects of therapeutics on both the host epithelium and bacterial survival. The development of this model required consideration of the delicate balance between HBE mucus accumulation (enough to benefit bacteria without affecting epithelial growth), antibiotic concentration (enough to prevent bacterial overgrowth without killing all bacteria), and the importance of the timing of *P. aeruginosa* inoculation relative to tobramycin addition. This novel bacterial–epithelial co-culture model demonstrated the importance of including bacteria in CF airway epithelial models for accurately determining the efficacy of ion transport-targeting drugs, and revealed that CFTR maturation and function are increased upon prolonged infection (24 h) with *P. aeruginosa*. This model can also be used with clinical isolates of *P. aeruginosa*, different bacterial species, e.g., *S. aureus*, and different antibiotics commonly administered to pwCF, such as amoxicillin and ceftazidime, in the presence and absence of HEMT. Although most pwCF have CFTR mutations that are approved for HEMT, there are still some pwCF with rare CFTR mutations that are left without CFTR-targeting drugs. This bacterial–epithelial co-culture model may reveal that HEMT is beneficial for these patients when tested in the presence of bacteria that enhance the HEMT-mediated rescue of CFTR. Furthermore, the use of bacteria and bronchial cells isolated from individuals with CF and other lung diseases will provide a personalized model consisting of bacteria and airway epithelia from the same individual, which can be employed to identify and optimize therapies.

## 5. Conclusions

This study introduces a co-culture model composed of primary human airway epithelia and prolonged *P. aeruginosa* infection. Using this model, we discovered that *P. aeruginosa* infection increased CFTR mRNA and protein production. Furthermore, *P. aeruginosa* infection during CFTR modulator therapy significantly improved CFTR function and led to a decrease in *P. aeruginosa* burden. These results highlight the importance of including metabolically active bacteria to accurately model the CF lung to test the efficacy of CFTR-targeting therapies in the context of CF lung infection, and to understand the effects of these therapies on bacterial burden in pwCF.

## Data Availability

The data that support the findings of this study are available from the corresponding authors upon reasonable request.
